# A color-digit Stroop task shows numerical influence on numerosity processing

**DOI:** 10.3758/s13421-024-01631-7

**Published:** 2024-09-11

**Authors:** Ronen Hershman, Lisa Beckmann‏, Eldad Keha, Michael Wagner, Liane Kaufmann, Avishai Henik

**Affiliations:** 1 Department of Psychology and The Zelman Center for Brain Science, Ben-Gurion University of the Negev, Beer-Sheva, Israel; 2 Department of Psychology, University of Innsbruck, Innrain 52f, 6020 Innsbruck, Austria; 3 Department of Psychology, Ludwig-Maximilians-Universität München, Munich, Germany; 4 Department of Psychology, The Hebrew University, Jerusalem, Israel; 5 Department of Psychology, Achva Academic College, Beer-Tuvia, Israel; 6 Department of Industrial Engineering & Management, Ariel University, Ariel, Israel; 7 Department of Neurology and Clinical Neuropsychology, Ernst von Bergmann Klinikum Potsdam, Potsdam, Germany

**Keywords:** Numerical cognition, Stroop effect, Numerosity, Cognitive control

## Abstract

The numerical Stroop task involves presenting participants with two digits that differ in physical size and numerical value and asking them to report which digit had the larger size or value while ignoring the other dimension. Previous studies show that participants have difficulty ignoring the irrelevant dimension and thus have implications on the automaticity of numerical processing. The present study investigates the automatic influence of numerical value on numerosity processing in a novel Stroop-like task. In two experiments, participants were presented with digits made of colored stripes and asked to identify the number of different colors. In both experiments, interference and facilitation effects were found, supporting the automaticity of symbolic number processing and its influence on numerosity processing. These findings expand upon previous research on numerical as well as counting Stroop tasks, and have potential implications for studying interference and basic numerical processing in children and clinical populations.

## Introduction

### The Stroop task

Cognitive control refers to our ability to inhibit habitual and automatic behavior and, instead, to execute a less-familiar task. One of the most well known tasks to examine cognitive control is the color-word Stroop task (Stroop, 1935). In this task, participants are presented with stimuli and are asked to report their presented color. Typically, there are mainly three different types of task conditions: congruent (e.g., the word RED written in red ink), incongruent (e.g., BLUE written in red), and neutral (the letter string XXXX). Usually and reliably, incongruent trials lead to significantly longer reaction times (RTs) than neutral trials, which is referred to as the *interference effect*. Additionally, congruent trials typically yield faster or similar RTs compared to neutral trials, thus reflecting a facilitation effect. Importantly, while the interference effect is large and robust, facilitation is usually smaller and more fragile (Hershman & Henik, 2019; MacLeod, 1991).

Since its first appearance in the mid-1930s, the Stroop task has evolved, and various Stroop-like tasks have been introduced and investigated (Glaser & Glaser, 1982; MacLeod, 1991; White, 1969). One of these versions is the numerical Stroop task (Besner & Coltheart, 1979; Henik & Tzelgov, 1982), which has been applied in the area of numerical cognition. In this task, participants are presented with two digits that differ in physical size and numerical value. Two versions of this task were introduced. In the first version of the task, participants are required to report which of the two digits has the physically larger size and ignore the numerical value (physical task). In the second version of the task, participants are requested to report the stimulus with the numerically larger value while ignoring the physical dimension (numerical task). The physical task (hereafter “the numerical Stroop task”) resembles the color-word Stroop task, as participants are supposed to attend to the physical dimension (i.e., the size of a stimulus or the ink color in the color-word Stroop task) while suppressing automatic processing (i.e., the numerical value of the digits in the numerical Stroop task or the word meaning in the color-word Stroop task). As in the color-word Stroop task, the numerical Stroop task consists of a congruent condition (the physically smaller digit is also numerically smaller), an incongruent condition (the physically smaller digit is numerically larger), and a neutral condition (equal numerical value but different in physical size). The results show the same response pattern as the color-word Stroop task, namely, interference and facilitation effects (e.g., Goldfarb & Henik, 2007; Henik & Tzelgov, 1982). Specifically, incongruent trials lead to longer RTs than neutral trials and we thus find an interference effect. Importantly, however, in the numerical Stroop task, congruent trials are commonly faster than neutral trials, thus producing a more reliable facilitation effect compared to this reported in the color-word Stroop task (Hershman & Henik, 2019; MacLeod, 1991).

Another numerical Stroop-like task was introduced by Bush et al. (1998). In their counting Stroop task, participants were required to report the number of presented stimuli that appeared on the screen. The stimuli could be repeated number words (e.g., one, two, three, four) that were incongruent with the number of stimuli presented or neutral repeated words (e.g., the word “bird” which was presented four times). In that study, an interference effect was found (i.e., slower RT for incongruent trials than for neutral trials). The main finding of the study was that the processing of the irrelevant dimension (“reading” of a number word) is automatic. However, since congruent trials were not included, it is not clear whether there was a facilitation effect or not.

### The present study

Similar to the color-word Stroop task (which shows that word reading is difficult to suppress for individuals with proficient reading skills), the numerical Stroop task (Henik & Tzelgov, 1982) as well as the counting Stroop task (Bush et al., 1998) suggests that the numerical values of the digits (or number words) are difficult to ignore, and, thus, are processed automatically by math-proficient individuals. In the present study, we set out to establish a novel Stroop-like task by varying the numerical value of the stimuli (i.e., single digits) as well as the number of colors that comprise the stimuli. Henceforth, the novel task is called the color-digit Stroop task. As in the numerical Stroop task, in the color-digit Stroop task the numerical value of the digits is the task-irrelevant dimension and, thus, should be ignored by participants. Rather, participants are asked to indicate the number of color stripes in a given digit. Thus, in our novel color-digit Stroop task, the number of colors constituting a stimulus is the relevant stimulus dimension. Because the numerical value of digits is highly salient and, thus, is processed automatically (as is the case in math-proficient individuals; e.g., Gómez et al., 2015; Rubinsten & Henik, 2005), we expected the task-irrelevant numerical values to modulate the processing of the task-relevant number of color stripes. In other words, a congruency effect due to the irrelevant stimulus dimension (i.e., the numerical value) on the task-relevant stimulus dimension (i.e., the number of color stripes comprising a digit) would further support the notion of the prepotent and automatic processing of the numerical value conveyed by a given digit (Henik & Tzelgov, 1982; MacLeod & MacDonald, 2000; Wood et al., 2009). Therefore, we hypothesized that the results would mimic those of the numerical Stroop task. Namely, congruent stimuli (e.g., the digit 4 consisting of four colors) will result in faster RTs compared to incongruent stimuli (e.g., the digit 4 consisting of two colors). We were able to confirm this hypothesis in Experiment 1 and again in Experiment 2, despite using stimuli with disrupted structural integrity. Overall, upon utilizing a novel Stroop-like variant, our findings disclose that the numerical processing of digits is a highly salient stimulus feature causing robust interference and facilitation effects, even when participants are asked to process another numerical stimulus dimension (i.e., the number of color stripes comprising a digit).

Please note that the relevant task here is counting. Accordingly, modulation of counting by the numerical values examines the automaticity of processing the counting or cardinality aspect of the digits. In contrast, the size-congruity effect (Henik & Tzelgov, 1982), mentioned above, examines the processing of the size or magnitude aspect of the digits. Since the numerical Stroop task and the color-digit task probe different aspects of the values of the symbolic system, they may present different results under specific situations or with different participants.

The color-digit task requires relatively little cognitive effort from participants as it requires neither reading skills (as is the case in the popular color-word Stroop task) nor a comparison process (as is the case in the popular numerical Stroop task, e.g., Besner & Coltheart, 1979; Henik & Tzelgov, 1982), and thus enables us to study interference processing without confounding factors of attention and/or reading fluency. Hence, this (language-independent) task could be used to study interference processing in pre-literate children and individuals (patients) with low-level/deficient reading.

## Experiment 1

### Method

#### Participants

Twenty participants (13 females and seven males, mean age 23.6 years, *SD* = 5.61) from Innsbruck University participated in the experiment in return for course credit. The number of participants was based on the number of participants (19) that were analyzed in our previous numerical Stroop study (Hershman et al., 2022). Since the anticipated effect size was expected to be relatively large (${\upeta }_{\text{p}}^{2}>0.7)$, and taking into account a dropping out of outliers, we invited 20 participants to take part in this experiment. The study was approved by the ethics committee of the Psychology Department. All participants were native German speakers and had no reported history of attention-deficit disorder, learning disabilities, or color blindness.

### Stimuli

Participants were presented with colored single-digit numbers (Arial font). The digits that were used were 1, 2, 3, and 4 in a size of 640 × 740 pixels. The single digit could consist of a different number of colors between one and four (red, blue, green, yellow; see examples of the stimuli in Table 1). Specifically, for each of the four color-number categories, the stimuli were cut horizontally into parts according to the required number of colors, and each horizontal part had the same height (in the case of three parts, there were two parts with a height of 247 pixels and one part – the middle one – with a height of 246 pixels). The number of colors could have been congruent with the numerical value of the presented colored single-digit number (e.g., the colored single-digit number 3 painted with three colors) or incongruent (e.g., the colored single-digit number 3 painted with two colors). In addition to the presented colored single-digit numbers, participants were also presented with colored rectangles of the same size and colors that served as the neutral condition. These types of stimuli have been suggested to activate less cognitive processing since the stimulus has no semantic/phonological/orthographical meaning (Hershman et al., 2021, 2022). The conditions and the stimuli for each participant were selected randomly from a pool that included all the possible combinations of stimulus in all possible color combinations, which yielded a set of 256 possible stimuli. The presented stimuli appeared against a white (RGB: 255, 255, 255) background.

**Table 1:**
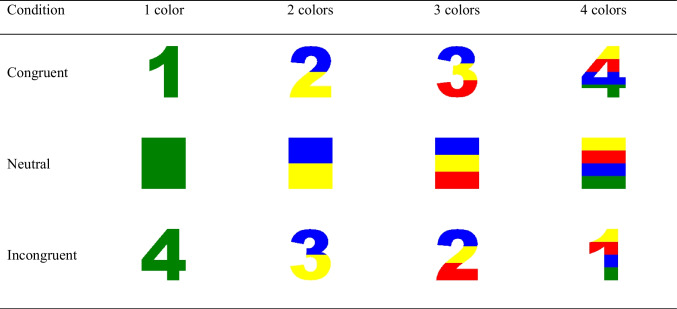
Examples of the presented stimuli in Experiment 1 (the entire set of stimuli can be downloaded from https://osf.io/bxhpy/?view_only=74b11af7662b4b2fac9700308e34dac3)

### Procedure

**Fig. 1:**
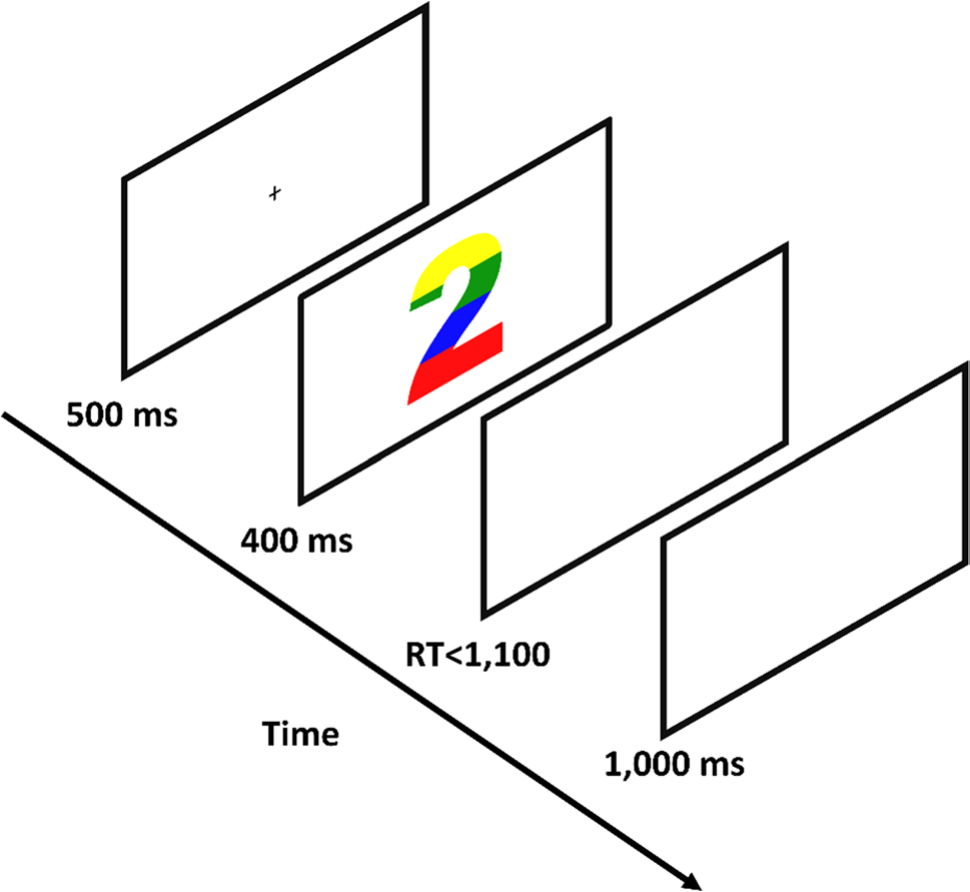
Example of a trial*.* Participants had to respond to the number of colors in the presented stimulus

Participants were tested online by using minnoJS (Zlotnick et al., 2015) on their own devices. The program required a spacebar response, ensuring participants only use computers rather than tablets or mobile phones. The experiment included 12 practice trials that were excluded from the analysis. After each practice trial, participants received feedback on their accuracy. Participants needed to achieve at least 80% correct trials in practice to proceed to the experimental part (i.e., at least ten correct responses). In the experimental part, participants carried out 432 experimental trials (144 for each congruency condition). At the beginning of each trial (see Fig. 1 for a visual demonstration) there was a black fixation cross in the center of the screen for 500 ms. The fixation was followed by a visual stimulus that appeared on the screen for 400 ms and was followed by a blank screen for a maximum of 1,100 ms or until a key-press. The times that were used in this experiment were in line with our previous color-word Stroop and Stroop-like tasks (Hershman et al., 2022; Hershman & Henik, 2019). This presentation time is a little bit shorter than the expected RTs (at least 400 ms). This fixed presentation time is important to avoid any perceptual differences that might occur due to an association of the presentation time with the RTs (i.e., presentation of the stimuli until the response). The trial ended with a 1,000-ms inter-trial interval (ITI) of a blank (white) screen. Participants were asked to press with a QWERTY keyboard the “Z” key (or the “Y” key in the case of a QWERTZ keyboard) if the stimulus consisted of one color, the “X” key if the stimulus consisted of two colors, the “N” key if the stimulus consisted of three colors, and the “M” key if the stimulus consisted of four colors (participants responded with two hands). This is a standard horizontal arrangement of responses that is used in general in color-word Stroop and Stroop-like tasks (Hershman et al., 2022; Hershman & Henik, 2019). RT was calculated from the appearance of the visual stimulus to the reaction in the form of a key-press.

### Results

Two participants were excluded from the analysis because they did not have a total success rate of 70%. For each participant (12 females and six males, mean age 23.11 years, *SD* = 5.19), mean RT and standard deviation were calculated separately across all the experimental trials. Then, extremely slow and fast responses were excluded from the analysis (i.e., RT larger or smaller than 2.5 z-scores from the mean of each subject).

Success rates for each participant in each condition were subjected to a two-way repeated-measures analysis of variance (ANOVA) with congruency (congruent, incongruent, and neutral) and the number of colors (1, 2, 3, and 4) as independent factors. Our analysis produced a meaningful ($B{F}_{10}\ge 3$) main effect for congruency, $F(2, 34)=66.84, p<.001, {\upeta }_{\text{p}}^{2}=.78, B{F}_{inc} > {10}^{5}$. Specifically, the success rate in incongruent trials (83.72%) was lower than in neutral trials (95.55%), $F\left(1, 17\right)=87.3, p < .001, BF_{10} > {10}^{4},$ and congruent trials (95.5%), $F\left(1, 17\right)= 68.95, p < .001, BF_{10} >\text{1,000}$. No difference was found between congruent and neutral trials, $F\left(1, 17\right)<1, p=.6, BF_{10} =.132$. In addition, no differences were found between the number of colors, $F\left(2, 34\right)<1, p=.65, {\upeta }_{\text{p}}^{2}=.032, B{F}_{inc}=.037,$ and there was no interaction between congruency and number of colors, $F\left(2, 34\right)<1, p=.69, {\upeta }_{\text{p}}^{2}=.037, B{F}_{inc}=.014$.

**Fig. 2:**
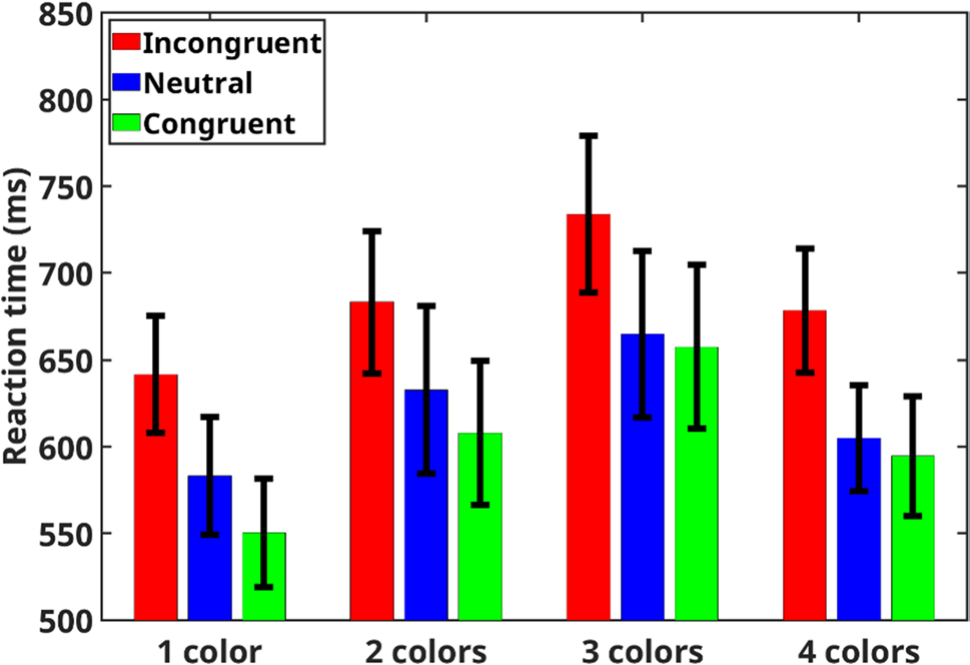
Mean reaction time for each congruency condition for each number of colors separately in Experiment 1. Error bars represent a 95% confidence interval around the mean

Mean RTs of correct response trials for each participant in each condition were subjected to a two-way repeated-measures ANOVA with congruency (congruent, incongruent, and neutral) and the number of colors (1, 2, 3, and 4) as independent factors (mean RTs in the various conditions are presented in Fig. 2). As expected, our analysis produced a meaningful ($B{F}_{10}\ge 3$) main effect for congruency, $F(2, 34)=194.2, p<.001, {\upeta }_{\text{p}}^{2}=.92, B{F}_{inc} > {10}^{34}$. Specifically, mean RT in incongruent trials was slower than in neutral trials, $F\left(1, 17\right)=173.88, p < .001, BF_{10} > {10}^{23}$, which was slower than in congruent trials, $F\left(1, 17\right)= 31.34, p < .001, BF_{10} > {10}^{4}$. Transitively, mean RT in incongruent trials was slower than in congruent trials, $F\left(1, 17\right)= 296.05, p< .001, B{F}_{10} > {10}^{31}$.

In addition, our analysis revealed a meaningful main effect for the number of colors, $F(3, 51)=34.71, p<.001, {\upeta }_{\text{p}}^{2}=.671, B{F}_{inc} > {10}^{31}$. Specifically, no clear differences in RT were found between two and four colors, $F\left(1, 17\right)= 2.56, p=.13, B{F}_{10}=2.03$. Mean RT for one color was faster than for four colors, $F \left(1, 17\right)= 27.19, p < .001, BF_{10} > {10}^{6},$ and for two colors was faster than for three colors, $F\left(1, 17\right)= 16.58, p<.001, BF_{10} > {10}^{6}$. Transitively, the mean RT for four colors was faster than for three colors, $F \left(1, 17\right)= 44.13, p < .001, BF_{10} > {10}^{11}$, and one color was faster than for both two colors, $F\left(1, 17\right)= 34.02, p<.001, BF_{10} > {10}^{9}$, and three colors, $F\left(1, 17\right)= 74.84, p<.001, BF_{10} > {10}^{15}$. Our analysis did not produce a clear interaction effect for congruency and number of colors, $F\left(6, 102\right)=2.85, p=.01, {\upeta }_{\text{p}}^{2}=.144, BF_{\text{inc}}=0.096$.

Notably, log transformation of the data led to the same results. Hence, any concerns about a skewed distribution effect on RTs could be ruled out (see the analysis of log-transformed data in the Appendix).

### Discussion

In the present experiment, RTs were slower in incongruent trials compared to neutral trials, which were slower than those of congruent trials. Similar results are frequently observed in the color-word Stroop task as well as in other Stroop-like tasks (MacLeod, 1991). In line with the results observed in the color-word Stroop task, the present results suggest that in incongruent trials, in addition to the information that is extracted from the colors themselves (the properties that participants are required to respond to), the numerical value of the stimuli (in the irrelevant dimension) is also processed. These results support our hypothesis that the irrelevant numerical value of the stimuli is difficult to ignore.

In contrast to the color-word Stroop task or to other language-based Stroop-like tasks, the color-digit Stroop task is language-independent. That is, the same task could be identically used in different languages and cultures. In Experiment 2, we aimed to replicate the results that were found with German-speaking participants using Hebrew-speaking participants. This replication would support the reliability as well as the cultural generalization of the task. In addition, with the aim of reducing the readability of the stimuli, which might decrease the interference from the irrelevant dimension (i.e., the numerical value of the stimuli), we used cut stimuli (Hershman, Sapir et al., 2024c). With these types of stimuli, we expected to improve the success rate (mainly of the incongruent trials). If the expected results are observed for RTs when no differences in accuracy are observed, no effect of accuracy on RTs could be ruled out.

## Experiment 2

In this experiment, we asked Hebrew-speaking participants to solve the color-digit Stroop task. While the task was the same as in Experiment 1 (participants were presented with colored stimuli and were required to evaluate how many colors the stimuli consisted of), the stimuli were more difficult to process because of the disruption of their integrity with gaps. Specifically, we removed five horizontal pieces from the stimuli (see examples of the stimuli in Table 2).

**Table 2:**
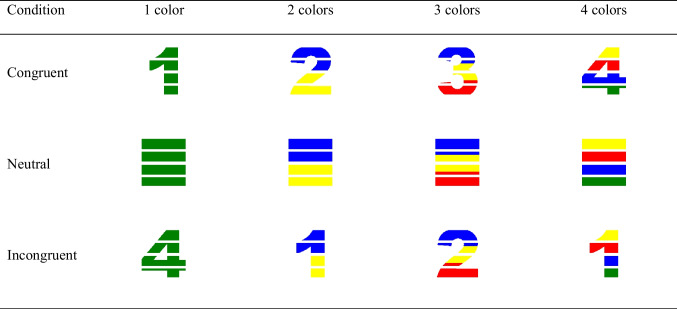
Examples of the presented stimuli in Experiment 2 (the entire set of stimuli can be downloaded from https://osf.io/bxhpy/?view_only=74b11af7662b4b2fac9700308e34dac3)

### Method

#### Participants

In line with the number of participants analyzed in Experiment 1, 18 participants (16 females and two males, mean age 23.13 years, *SD* = 1.29) from Ben-Gurion University of the Negev participated in the experiment in return for course credit. The study was approved by the ethics committee of the Psychology Department. All participants were native Hebrew speakers and had no reported history of attention-deficit disorder, learning disabilities, or color blindness.

#### Stimuli

The stimuli in Experiment 2 were identical to those in Experiment 1, with one important difference. Here, all the presented stimuli were cut into four pieces. This cutting was done by removing five pieces from the stimuli. Specifically, the upper and lower 20 pixels were removed from the stimuli, as well as the pixels between 166 and 205, 351 and 390, and 536 and 575 (see examples of the stimuli in Table 2).

#### Procedure

The procedure for Experiment 2 was identical to that of Experiment 1.

### Results

Success rates for each participant in each condition (96.39%, 94%, and 88% for congruent, neutral, and incongruent trials, respectively) were subjected to a two-way repeated-measures ANOVA with congruency and the number of colors as independent factors. Our analysis produced no congruency effect, $F\left(2, 34\right)<1, p=.57,{\upeta }_{\text{p}}^{2}=.03, B{F}_{inc}=.06$, no differences between number of colors, $F\left(2, 34\right)<1, p=.58,{\upeta }_{\text{p}}^{2}=.04, B{F}_{inc}=.1,$ and no interaction between congruency and number of colors, $F\left(2, 34\right)<1, p=.71, {\upeta }_{\text{p}}^{2}=.035, B{F}_{inc}=.1$.

**Fig. 3:**
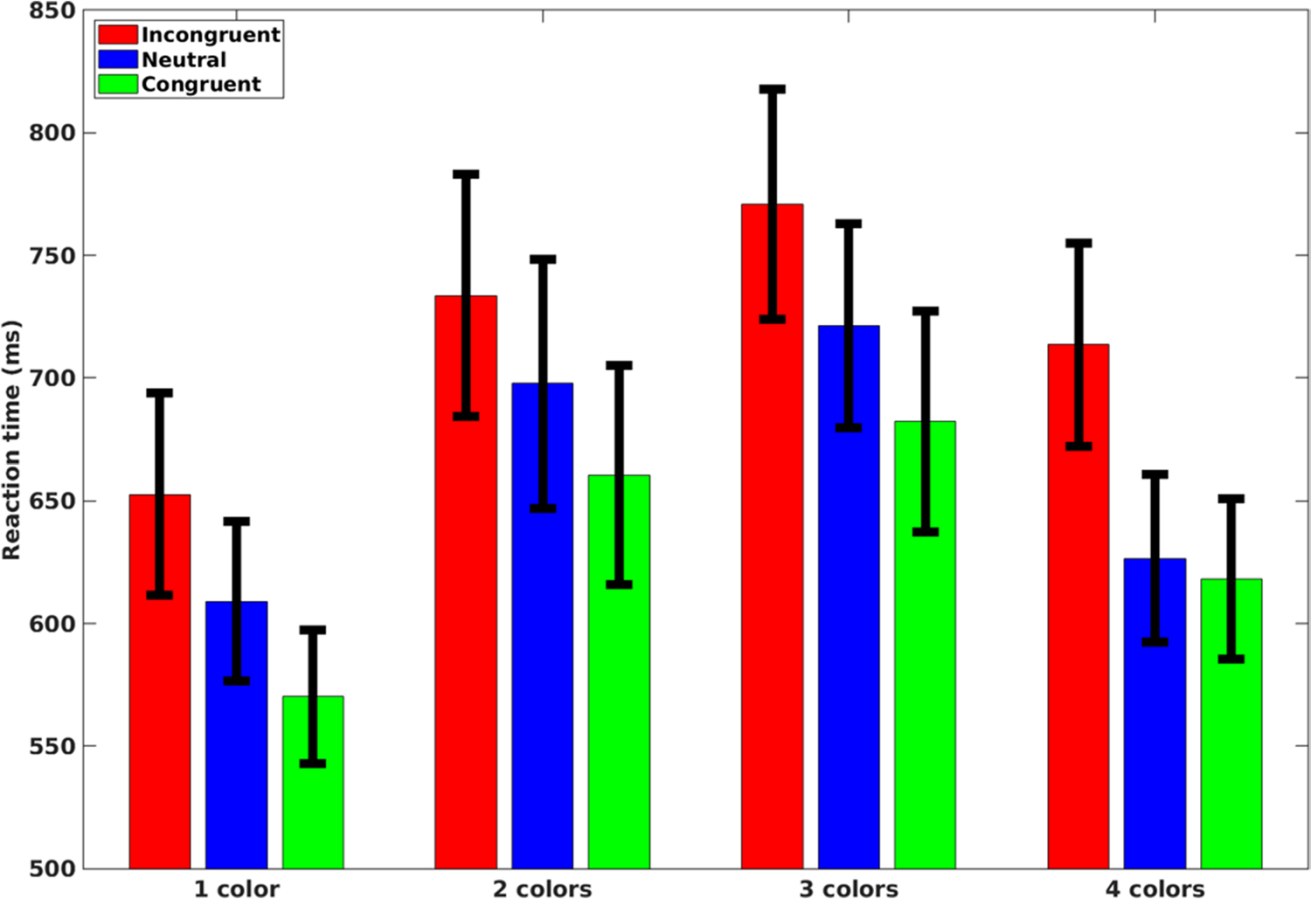
Mean reaction time for each congruency condition for each number of colors separately in Experiment 2. Error bars represent a 95% confidence interval from the mean

Similar to Experiment 1, mean RTs of correct trials for each participant in each condition were subjected to a two-way repeated-measures ANOVA (the exclusion criteria of RTs were similar to those of Experiment 1, and all participants had a total success rate above 70%) with congruency (congruent, incongruent, and neutral) and the number of colors (1, 2, 3, and 4) as independent factors (mean RTs in the various conditions are presented in Fig. 3). As expected, our analysis produced a meaningful main effect for congruency, $F\left(2, 34\right)=128.6, p<.001, {\upeta }_{\text{p}}^{2}=.88, B{F}_{inc} > {10}^{13}$. Specifically, mean RT in incongruent trials was slower than in neutral trials, $F\left(1, 17\right)=129.43, p < .001, BF_{10} > {10}^{16},$ which was slower than in congruent trials, $F\left(1, 17\right)= 49.01, p < .001, BF_{10} > {10}^{7}$. Similar to Experiment 1, mean RT in incongruent trials was also slower than in congruent trials, $F\left(1, 17\right)= 163.3, p< .001, B{F}_{10} > {10}^{26}$.

In addition, our analysis produced a meaningful main effect for the number of colors, $F\left(3, 51\right)=42.651, p<.001, {\upeta }_{\text{p}}^{2}=.72, B{F}_{inc} > {10}^{13}$. Mean RT for one color was faster than for four colors, $F\left(1, 17\right)=48.88, p< .001, B{F}_{10}> {10}^{7}$. Mean RT for four colors was faster than for two colors, $F\left(1, 17\right)=13.49, p=.001, BF_{10}>\text{1,000},$ and mean RT for two colors was faster than for three colors, $F\left(1, 17\right)= 10.08, p=.005, BF_{10}=233.111.$ Transitively, the mean RT for four colors was faster than for three colors, $F \left(1, 17\right)= 43.65, p < .001, BF_{10} > {10}^{11}$, and mean RT for one color was faster than for both two colors, $F\left(1, 17\right)= 45.3, p<.001, BF_{10} > {10}^{12}$, and three colors, $F\left(1, 17\right)= 79.53, p<.001, BF_{10} > {10}^{17}$. Moreover, our analysis tended to produce a meaningful interaction effect for congruency and the number of colors, $F\left(6, 102\right)=5.39, p<.01, {\upeta }_{\text{p}}^{2}=.24, B{F}_{inc}=1.986$. Post hoc analysis suggested that while there was both facilitation and interference for one to three colors, there was only interference when there were four colors in the relevant dimension (see Table 3 for full statistical results).

**Table 3:** Statistical results for the interaction in Experiment 2

Comparison	1 color	2 colors	3 colors	4 colors
Incongruent vs. neutral	*F*(1, 17) = 43.8	*F*(1, 17) = 22.61	*F* (1, 17) = 44.44	*F*(1, 17) = 99.46
	*p* < .001	*p* < .001	*p* < .001	*p* < .001
	*BF*_*10*_ >1,000	*BF*_*10*_ = 164	*BF*_*10*_ >1,000	*BF*_*10*_ > 10^5^
Neutral vs. congruent	*F*(1, 17) = 43.8	*F*(1, 17) = 16.28	*F*(1, 17) = 14.69	*F*(1, 17) = 1.9
	*p* < .001	*p* < .001	*p* = .001	*p* = .19
	*BF*_*10*_ >1,000	*BF*_*10*_ = 42	*BF*_*10*_ = 29.12	*BF*_*10*_ = 0.547
Incongruent vs. congruent	*F*(1, 17) = 88.7	*F*(1, 17) = 94.89	*F*(1, 17) = 118.06	*F*(1, 17) = 84.74
	*p* < .001	*p* < .001	*p* < .001	*p* < .001
	*BF*_*10*_ > 10^5^	*BF*_*10*_ > 10^5^	*BF*_*10*_ > 10^6^	*BF*_*10*_ > 10^5^

Similar to Experiment 1, log transformation of the data led to the same results. Hence, any concerns about a skewed distribution effect on RT should be ruled out (see the analysis of log-transformed data in the Appendix).

### Discussion

Similar to Experiment 1, RTs were slower in incongruent trials compared to neutral trials, which were slower than those of congruent trials. These results suggest that in addition to the interference that is raised due to the contradicting pieces of information in the incongruent trials, there is also facilitation that occurs due to the supporting information that is provided by the task-irrelevant stimulus properties. These two effects (i.e., both interference and facilitation) support the hypothesis that the irrelevant numerical value of the stimuli is being processed automatically. In contrast to Experiment 1, no differences in accuracy were found between the congruency conditions. Therefore, the difference in RTs between the conditions cannot be associated with differences in accuracy.

One could argue that the cutting of the stimuli into four pieces might cause potential congruency of the number of colors with the number of stripes. That is, when participants are asked to respond to the number of colors, they might (hypothetically) respond to the number of stripes (in addition to responding to the number of colors). This congruency could be an alternative explanation for the observed results. However, RTs of the neutral stimuli with four colors tended to be identical to those with one color, $B{F}_{10}=0.428\equiv B{F}_{01}=2.336$. If there was an influence of congruency between the number of colors and the number of pieces, the facilitation of four colors should have been observed. Therefore, this potential artifact is less likely. Moreover, in four colors, no facilitation (i.e., faster responses for congruent trials than for neutral trials) was found. Hence, it is unlikely that participants tended to respond to the number of stripes. Please note that this absence of facilitation does not influence the general pattern of the results (i.e., both congruency and interference effects for one to four colors).

## General discussion

In the present study, we conducted two experiments (with both German- and Hebrew-speaking participants) using a novel Stroop-like task. We showed that the irrelevant dimension (the numerical value of a symbolic number) strongly influenced the processing of the relevant dimension (numerosity, here of colors). This was true for regular symbolic numbers (Experiment 1) as well as numbers that were disrupted by gaps (Experiment 2).

We found a congruency effect: responses to congruent trials were faster than to incongruent trials. This provides evidence for the automaticity of symbolic number processing and its influence on the processing of numerosity. These results are in line with results that were found in the past with the numerical Stroop task (Henik & Tzelgov, 1982; Hershman et al., 2022) that showed numerical values of digits are processed automatically. In addition to the congruency effect, we also found a facilitation effect: responses to congruent trials were faster than to neutral trials. This facilitation, as well as the interference (i.e., slower responses for incongruent than for neutral trials), makes the findings clearer and more reliable; that is, there is automatic processing of the task-irrelevant properties (numerical value of the presented symbolic number). This processing interferes with our response when it is incongruent with the response to the task-relevant properties, and it facilitates our response when it is congruent with the response to the task-relevant properties.

Another Stroop-like task variant with resembling results is the counting Stroop used by Bush et al. (1998). In this task, participants were presented with a set of one to four identical words on a screen and were asked to indicate the number of items. The words were neutral (i.e., “dog” written four times) or incongruent (i.e., “two” written four times). Similar to our study, as in Bush et al.’s study, an interference effect was found. The main finding of this study was that the processing of the irrelevant dimension (“reading” of a word number) is automatic. Our results extend these findings. Namely, the effect is not only reflected in the counting of symbols but also in other properties (in our case, the colors) of the number.

While counting is required in both the counting Stroop and the color-digit Stroop tasks, the two tasks are different in the number of presented objects. In the counting Stroop task utilized by Bush et al. (1998), participants were required to report the presented number of objects on the screen, with stimuli ranging from one to four number words (“one,” “two,” “three,” “four” that were incongruent with the number of stimuli presented) or neutral words (e.g., “bird”). In our task, participants were presented with only one single stimulus (i.e., Arabic digits and squares printed in different color stripes). Thus, the counting Stroop task used by Bush et al. was visually more complex than our task. Moreover, the two tasks are different in the complexity level of the irrelevant dimension of the stimuli. While the counting Stroop task employed by Bush et al. used (number) words as an irrelevant stimulus dimension (which means that reading fluency was a prerequisite to obtaining the expected RT effects), the irrelevant stimulus dimension in our task consisted of single digits (that did not require word reading). Hence, the mental effort that was required for the processing of the irrelevant dimension in Bush et al.’s study was higher than in our task. Using Arabic digits (which for skilled calculators are highly overlearned and processed automatically) and color (in the absence of color blindness) imposes little cognitive effort as no reading skills are required but rather visual differentiation processes are drawn upon.

In the popular numerical Stroop task (Henik & Tzelgov, 1982), participants are required to decide which of two simultaneously presented Arabic digits is the physically larger one. In the color-digit Stroop task in our study, only one Arabic digit (composed of color stripes) is presented at a time, requiring participants to indicate the number of color stripes. Thus, the color-digit Stroop task is visually less complex (does not require the visual inspection of two horizontally presented stimuli that require saccadic movements) as the Arabic single digit is presented in the middle of the screen, and the stimulus does not require a comparison of two simultaneously presented stimuli. As a result, the color-digit Stroop task places less load on (visual-) attentional processes.

In the present study, we chose to use digits between 1 and 4 with the aim of using a reasonable number of responses. That is, for each number of colors, one response key was used. In addition, with the aim of avoiding a complicated counting of the number of colors, we preferred to keep the number of colors in the subitizing range (thus ensuring that the number of colors would be enumerated quickly and efficiently). However, it is not clear whether the effect is limited to the tested range or not. In a further study (Hershman, Keha, Sapir et al., 2024b), participants were asked to decide whether the number of presented colors (1–9 excluding 5) was smaller or larger than 5. Also, in that experiment, there were congruent, incongruent, and neutral trials. In line with the results of the present study, as in that study, both interference and facilitation effects were found. Therefore, we tend to believe that the congruency effect is valid and reliable.

Further studies that will examine a larger number of colors (i.e., 1–9) with a vocal response will solve the issue of the number of responses (i.e., too many response keys yielding a higher cognitive load and a more difficult response selection in case of manual responses) and might answer this question explicitly.

Interestingly, the further comparison to the five-color color-digit Stroop task (Hershman, Keha, Sapir et al., 2024b) also allowed us to examine the source of interference and to test whether the interference in the number of colors stems mostly from the fact that two responses compete on the same stimulus (i.e., response conflict) or if it mostly depends on the semantic processing of the irrelevant digits (i.e., semantic conflict). In line with previous studies that compared these conflicts in the classic color-word Stroop paradigm (De Houwer, 2003; Hershman & Henik, 2020; Shichel & Tzelgov, 2018), both response and semantic conflicts were found. These results suggest that when participants are requested to respond to a number of colors, semantic processing of their numerical value is difficult to inhibit. Therefore, we believe that both the interference and the facilitation that were found in the present study are associated with the processing of the numerical value of the stimuli (that caused semantic conflict) in addition to a potential response conflict.

It is important to notice that the larger the number of stripes, the smaller the area, as the size of the stimuli (in pixels) did not change. However, while the size of the colored areas might explain why responses for one color were faster than for two colors, which in turn were faster than for three colors, it cannot explain the observed congruency effects (e.g., the number of pixels for each color cannot explain why when only one color was presented, the responses were faster if the presented digit was “1” (the congruent condition), than if it was “4” (the incongruent condition)). Therefore, we believe that the number of pixels as a function of the number of colors cannot explain the observed effects.

Interestingly, RTs for one and four colors were significantly faster than for both two and three colors. In the interview after the experiment (that aimed to gather information about participants' strategies upon solving the novel color-digit Stroop task), our participants consistently reported that both one and four colors were the easiest to detect. Most of them reported that when only one color was presented, no counting was required (and the estimation was made easily). In the same line, most of the participants reported that when four colors were presented, there was no doubt regarding the number of colors (simply because the maximal number of colors appeared). Therefore, as reflected in our participants’ reports, the fast RTs for both one and four colors and the relatively slow RTs for both two and three colors were caused by an end effect. In a further study (Hershman, Keha, Sapir et al., 2024b) that included more colors (up to nine colors), this end effect was confirmed since four colors did not show faster responses than for either two or three colors.

The present task also adds to the commonly used numerical Stroop task (Henik & Tzelgov, 1982). The numerical Stroop examines the relationship between symbolic numbers and magnitude or size, whereas the present color-digit Stroop task examines the relationship between symbolic numbers and counting or cardinality. Because these two tasks probe different aspects of the values of the symbolic system, they may present different results under specific situations or with different participants. For example, children in kindergarten or early first grade of school may present a congruity effect in the color-digit Stroop task but not in the numerical Stroop task. Rubinsten et al. (2002) conducted a cross-sectional study of the numerical Stroop task (Henik & Tzelgov, 1982) in elementary school. They found that early in first grade, children were familiar with the symbolic system but did not present a size-congruity effect. That is, in the physical task (when numerical values were irrelevant), they presented no size-congruity effect. A similar group of children or children in kindergarten would be expected to show a similar pattern in the size-congruity task in the face of a significant congruity effect in the present color-digit Stroop task. It is plausible to speculate that due to early experience with counting, these young children might associate the numerical symbols with counting or even cardinality, yet may not have associated the symbols with size or magnitude in general. The latter, the association between the symbolic system and size, is the driving force behind the numerical Stroop task.

Together, previous and current results elaborate on our understanding of the processing of automaticity of numerical values in general and of numerical values of symbolic numbers in particular. Importantly, the understanding gained here, which is related to cognitive control and number processing, could be applied to other areas, such as the acquisition of mathematical skills, dyscalculia, and acalculia. Further studies using special populations (e.g., with dyscalculia and acalculia), as well as developmental studies, might improve our knowledge about the mechanisms behind it and, moreover, might be used as a diagnostic tool for mathematical disabilities/difficulties.

Recent research has suggested that the color-word Stroop and Stroop-like effects are due to two conflicts rather than one (Littman et al., 2019). One conflict, referred to as the information conflict, is due to the contradicting pieces of information provided by the stimuli (ink color and word meaning in the color-word task and number of colors and numerical value in the present experiments). A second conflict, referred to as the task conflict, is due to a competition between a task naturally associated with the stimuli (reading words in the color-word task and processing numerical values in the present experiments) and the task required in the experiment (naming color or counting colors). It has already been suggested that task conflict is the primary component of the Stroop effect because informational conflict can only arise after engaging in an irrelevant task (Levin & Tzelgov, 2014). However, the present design enables us to examine the information conflict but not the task conflict. Hence, it is not clear how the two conflicts (information and task conflicts) underlie the inability to ignore the irrelevant task or dimension. Future studies should address this issue by designing experiments that are capable of disentangling the two types of conflicts or by using other markers that can assess the different conflicts (Hershman, Keha, Beckmann, et al., 2024a). One such marker is pupil dilation (Hershman & Henik, 2020). Interestingly, pupil dilation has been suggested to be an efficient temporal measure for cognitive effort in general and task conflict in particular. Another approach to examining task conflict for number processing is to test the effect of automatic number processing under conditions of reduced cognitive control (Goldfarb & Henik, 2007).

## Conclusion

In two experiments, we employed two different versions of a novel Stroop-like task. In the first experiment, we looked at the influence of automatic symbolic numerical processing on the task of numerosity processing. In particular, participants had to count the number of colors in a given stimulus and ignore the numeral itself. We found strong interference and facilitation effects, implicating strong automaticity influencing the responses on the numerosity performance. In the second experiment, we disrupted the structural integrity of the numbers with gaps and were still able to replicate the effects, underscoring the strength of the numerical dimension only with prolonged RTs overall. Consequently**,** we have provided evidence for the influence of numerical processing on the strong, evolutionarily important, numerosity processing.

To conclude, the novel color-digit Stroop task requires relatively little cognitive effort from participants as it neither requires reading skills (as is the case in the popular color-word Stroop task) nor a comparison process (as is the case in the popular numerical Stroop tasks – e.g., Besner & Coltheart, 1979; Henik & Tzelgov, 1982 – which imposes higher loads on attentional processes) and thus enables us to study interference processing without confounding factors of reading fluency and/or attention. In the present study, the same experiment was conducted with both German- and Hebrew-speaking participants and showed the same results (i.e., both interference and facilitation effects). Hence, the presented task has been shown to yield language- (and culture-) independent robust results and could be used to study interference processing in pre-literate children and individuals (patients) with low/deficient reading.

## Data Availability

The data, as well as the stimuli of the experiments, can be retrieved via the Open Science Framework at: https://osf.io/bxhpy/?view_only=74b11af7662b4b2fac9700308e34dac3.

## Appendix

### Analysis of log-transformed reaction times (RTs)

#### Experiment 1

Our analysis produced a meaningful ($B{F}_{10}\ge 3$) main effect for congruency, $F\left(2, 34\right)=210.97, p<.001, {\upeta }_{\text{p}}^{2}=.925, B{F}_{inc} > {10}^{39}$. Specifically, mean RT in incongruent trials was slower than in neutral trials, $F\left(1, 17\right)=181.39, p < .001, BF_{10} > {10}^{24}$, which was slower than in congruent trials, $F\left(1, 17\right)= 37.58, p < .001, BF_{10} > {10}^{5}$. Transitively, mean RT in incongruent trials was slower than in congruent trials, $F\left(1, 17\right)= 330.23, p< .001, B{F}_{10} > {10}^{31}$.

In addition, our analysis revealed a meaningful main effect for the number of colors, $F\left(3, 51\right)=43.95, p<.001, {\upeta }_{\text{p}}^{2}=.72, B{F}_{inc} > {10}^{34}$. While no clear differences in RT were found between two and four colors, $F\left(1, 17\right)= 2.42, p=.14, B{F}_{10}=1.47$, mean RT for one color was significantly faster than for four colors, $F \left(1, 17\right)= 31.84, p < .001, BF_{10} >{10}^{6},$ and mean RT for two colors was faster than for three colors, $F\left(1, 17\right)= 19.9, p<.001, BF_{10} >{10}^{6}$. Transitively, the mean RT for four colors was faster than for three colors, $F \left(1, 17\right)= 50.03, p < .001, BF_{10} >{10}^{11}$, and mean RT for one color was faster than for both two colors, $F\left(1, 17\right)= 50.01, p<.001, BF_{10} >{10}^{11}$, and three colors, $F\left(1, 17\right)= 99.54, p<.001, BF_{10} >{10}^{17}$. Overall, our analysis did not produce a clear meaningful interaction effect for congruency and number of colors, $F\left(6, 102\right)=4.379, p=.005, {\upeta }_{\text{p}}^{2}=.205, BF_{\text{inc}}=0.39$.

#### Experiment 2

Our analysis produced a meaningful main effect for congruency, $F\left(2, 34\right)=157.93, p<.001, {\upeta }_{\text{p}}^{2}=.903, B{F}_{inc} > {10}^{30}$. Specifically, mean RT in incongruent trials was slower than in neutral trials, $F\left(1, 17\right)= 161.63, p < .001, BF_{10} > {10}^{16},$ which was slower than in congruent trials, $F\left(1, 17\right)= 58.04, p < .001, BF_{10} > {10}^{8}$. Similar to Experiment 1, mean RT in incongruent trials was slower than in congruent trials, $F\left(1, 17\right)= 208.72, p< .001, B{F}_{10} > {10}^{29}$. In addition, our analysis produced a meaningful main effect for the number of colors, $F\left(3, 51\right)=40.43, p<.001, {\upeta }_{\text{p}}^{2}=.74, B{F}_{inc} > {10}^{38}$. Mean RT for one color was faster than for four colors, $F\left(1, 17\right)=50.7, p< .001, B{F}_{10}> {10}^{8}$, mean RT for four colors was faster than for two colors, $F\left(1, 17\right)=13.89, p=.001, BF_{10}>\text{1,000},$ and mean RT for two colors was faster than for three colors, $F\left(1, 17\right)= 16.32, p<.001, BF_{10}>\text{1,000}.$ Transitively, the mean RT for four colors was faster than for three colors, $F \left(1, 17\right)= 48.96, p < .001, BF_{10} > {10}^{11}$, and mean RT for one color was faster than for both two colors, $F\left(1, 17\right)= 49.75, p<.001, BF_{10} > {10}^{13}$, and three colors, $F\left(1, 17\right)= 92.57, p<.001, BF_{10} > {10}^{18}$. Moreover, our analysis tended to produce a meaningful interaction effect for congruency and the number of colors, $F\left(6, 102\right)=7.3, p<.001, {\upeta }_{\text{p}}^{2}=.3, B{F}_{inc}=2.24$.
